# Quantitative measurement of IgG to SARS‐CoV‐2 antigens using monoclonal antibody‐based enzyme‐linked immunosorbent assays

**DOI:** 10.1002/cti2.1369

**Published:** 2022-01-30

**Authors:** Ingrid Sander, Sabine Kespohl, Eva Zahradnik, Philipp Göcke, Ingolf Hosbach, Burkhard L Herrmann, Thomas Brüning, Monika Raulf

**Affiliations:** ^1^ Institute for Prevention and Occupational Medicine of the German Social Accident Insurance Institute of the Ruhr University Bochum (IPA) Bochum Germany; ^2^ Practice for Laboratory Medicine and Microbiology Bochum Bochum Germany; ^3^ BG University Hospital Bergmannsheil Bochum Bochum Germany; ^4^ Division of Endocrinology & Laboratory Research Bochum Germany

**Keywords:** COVID‐19, IgG, nucleocapsid, parallelism, quantitative enzyme‐linked immunosorbent assay, repeatability, Spike‐S1 protein, standardisation

## Abstract

**Objective:**

Standardised quantitative analysis of the humoral immune response to SARS‐CoV‐2 antigens may be useful for estimating the extent and duration of immunity. The aim was to develop enzyme‐linked immunosorbent assays (ELISAs) for the quantification of human IgG antibodies against SARS‐CoV‐2 antigens.

**Methods:**

Enzyme‐linked immunosorbent assays were developed based on monoclonal antibodies against human IgG and recombinant SARS‐CoV‐2 antigens (Spike‐S1 and Nucleocapsid). The WHO 67/086 immunoglobulin and WHO 20/136 SARS‐CoV‐2 references were used for standardisation. Sera of a study group of COVID‐19‐positive subjects (*n* = 144), pre‐pandemic controls (*n* = 135) and individuals vaccinated with BioNTech–Pfizer BNT162b2 vaccine (*n* = 48) were analysed. The study group sera were also tested using EuroImmun SARS‐CoV‐2‐ELISAs and a quantitative S1‐specific fluorescence enzyme immunoassay (FEIA) from Thermo Fisher.

**Results:**

The ELISA results were repeatable and traceable to international units because of their parallelism to both WHO references. In the study group, median anti‐S1‐IgG concentrations were 102 BAU mL^−1^, compared to 100 and 1457 BAU mL^−1^ in the vaccination group after first and second vaccination, respectively. The ELISAs achieved an area under the curve (AUC) of 0.965 (S1) and 0.955 (Nucleocapsid) in receiver operating characteristic (ROC) analysis, and a specificity of 1 (S1) and 0.963 (Nucleocapsid) and sensitivity of 0.903 (S1) and 0.833 (Nucleocapsid) at the maximum Youden index. In comparison, the commercial assays (S1‐FEIA, S1 and Nucleocapsid ELISA EuroImmun) achieved sensitivities of 0.764, 0.875 and 0.882 in the study group, respectively.

**Conclusions:**

The quantitative ELISAs to measure IgG binding to SARS‐CoV‐2 antigens have good analytical and clinical performance characteristics and units traceable to international standards.

## Graphical abstract

We developed ELISAs to quantify IgG levels in response to SARS‐CoV‐2 antigens with good analytical and clinical performance that can be carried out using common laboratory equipment, provide results in units traceable to international standards (IU and BAU) and can be easily adapted to quantify IgG to SARS‐CoV‐2‐mutated proteins and other antigens.

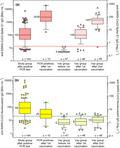

## Introduction

Specific detection of the immune response to SARS‐CoV‐2 infection plays an important role in tracking the spread of COVID‐19 in the population.^1^, ^2^, ^3^, ^4^, ^5^, ^6^, ^7^ IgG antibodies in particular remain detectable after the end of the disease or even after infections without symptoms.^8^, ^9^ Numerous immunoassays have been developed to detect IgG to the SARS‐CoV‐2 antigens,^10^, ^11^ some of which are characterised by high specificity and sensitivity.^12^, ^13^, ^14^, ^15^, ^16^ Both the spike protein that binds to the receptors on the host cell for virus entry and the nucleocapsid protein required for virus replication are recognised by human antibodies and serve as antigens for the serological detection of infection.^17^, ^18^ Normally, the nucleocapsid protein is present in cells at higher copy numbers than the spike protein^19^ and is responsible for the high sensitivity of immunoassays based on this antigen due to its high immunogenicity. The spike protein is located on the surface of the enveloped RNA virus and consists of trimers of two glycosylated subunits (S1 and S2). The S1 subunit contains the receptor‐binding region (RBD) and shows less homology to other coronaviruses than the S2 protein.^20^ Because antibodies with proven neutralising activity bind to the spike protein and the RBD in particular,^21^, ^22^, ^23^ the spike protein is also the major antigen target for vaccines. After the first vaccination, antibody responses are elicited and then boosted with the second vaccine dose to protein‐binding IgG levels above those of human convalescent sera.^24^, ^25^ Specific IgG antibody titres decrease over time but remain elevated up to 6 months after a complete vaccination.^26^, ^27^ The long‐term persistence of SARS‐CoV‐2‐specific antibodies is unclear as is whether the presence of antibodies confers protective immunity against SARS‐CoV‐2.^28^

As the vaccination campaign progresses and breakthrough infections occur even after full vaccination,^29^ immunoassays for the quantitative detection of anti‐SARS‐CoV‐2 antibodies are becoming increasingly import. On the one hand, a significant increase in antibody concentration indicates successful vaccination; on the other hand, the antibody level may indicate the probability and duration of immune protection and sterilising immunity. Neutralising antibody titres were found to be strongly associated with the magnitude of the IgG response^30^, ^31^, ^32^, ^33^ and were highly predictive of the immune protection from symptomatic SARS‐CoV‐2 infection.^34^

Quantitative immunoassays are part of the standard repertoire in serology diagnostics and research. For example, the quantification of antigen‐specific human IgG antibodies is an important component in the diagnosis of hypersensitivity pneumonitis.^35^ Antigen‐specific IgG concentrations are determined using a calibration curve traceable to international human immunoglobulin standards,^36^ thus enabling values to be compared across laboratories if the same antigens and assay components are used. In the case of anti‐SARS‐CoV‐2 immunoglobulin, an international standard now exists^37^ that has been evaluated in a WHO collaborative study.^38^

The aim of this study was to develop quantitative enzyme‐linked immunosorbent assays (ELISA) in order to detect the binding of human IgG to the Spike‐S1 and nucleocapsid virus proteins. The measurement of antibodies against both immunodominant SARS‐CoV‐2 antigens usually allows for the differentiation of the immune response after vaccination and infection, as almost all licenced vaccines target the spike protein. In order to validate the developed ELISAs, a study group of individuals with positive SARS‐CoV‐2 PCR test results (true positives) and a control group with sera taken before the COVID‐19 pandemic (true negatives) were used for ROC analysis. The results of the study group using our newly developed ELISAs were compared to those of commercially available anti‐SARS‐CoV‐2 IgG immunoassays from EuroImmun, Lübeck, Germany and Thermo Fisher Scientific, Darmstadt, Germany. For standardisation and to calculate conversion factors, international WHO references for immunoglobulins^36^ and SARS‐CoV‐2‐specific antibodies^37^ were used. Finally, sera of vaccinated individuals were tested with the new ELISAs before and after vaccination.

## Results

### Study and control groups

Sera classified as positive by PCR test (study group) and pre‐pandemic sera (control group) were used for immunoassay development and validation (Table 1). The participants in the study group were older than those in the control group (mean difference was 8 years).

**Table 1:** Basic data on the study group and the controls

	Study group	Control group
	*n* = 144	*n* = 135
Age (years)	18–82 [Median = 50]	18–73 [Median = 40]
Gender		
Male (%)	37% (53/144)	32% (43/135)
Female (%)	63% (91/144)	68% (92/135)
Sample collection dates	3/2020–2/2021	4/2006–12/2018
SARS‐CoV‐2 PCR positivity	100%	
Days after positive PCR test at collection	14–96 [Median = 39]	
Symptomatic	91	
Asymptomatic	4	
Unknown	49	

### Assay design and analytical performance

**Figure 1:**
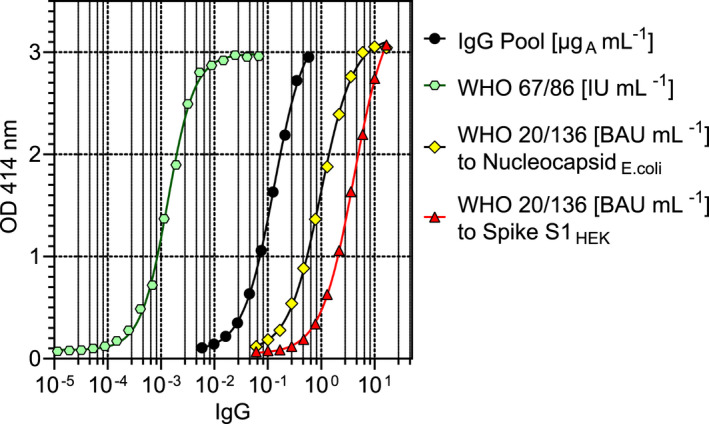
Reference curves of the IgG ELISA. Relationship between optical density (OD) and IgG concentration of the IgG pool serum used as a laboratory reference, and the WHO Ig (IU, International units) and SARS‐CoV‐2 reference (BAU, Binding activity units) preparations in the ELISA. Binding of the latter to the nucleocapsid expressed in *E. coli* resulted in higher OD values than binding to the Spike‐S1 subunit expressed in a human embryonal kidney (HEK) cell line at same dilutions.

For the quantification of human IgG, well‐characterised monoclonal antibodies were used, which bind to all IgG subclasses with high specificity and affinity.^39^ A biotinylated version of mAb HP6017, which binds to the Fc part of human IgG, was used as the detection antibody. For the reference curve, the capture antibody mAb HP6045 was used, which binds to the Fd part of IgG. A steep sigmoidal curve with the inflection point at approximately 0.12 µg_A_ mL^−1^ was obtained using a pool serum with known IgG concentration. The dilution series of the WHO 67/086 Immunoglobulin (Ig) reference preparation and the WHO 20/136 SARS‐CoV‐2 reference binding to the Spike‐S1_HEK_ or Nucleocapsid*_E. coli_* antigens resulted in parallel curves (Figure 1). The WHO 20/136 reference pool showed higher binding to the Nucleocapsid compared to the Spike‐S1 antigen. Parallelism to the reference curve was controlled by the intra‐assay coefficient of variation (CV). The CV% of repeated experiments and of the study groups' dilution series are presented in Table 2. The intra‐assay CV for the WHO references dilution series was 5% or lower, and the inter‐assay CV was below 8%. Because of the parallelism of the curves, conversion factors could be calculated (Table 3). The WHO 67/87 Ig reference was measured to contain 8482 µg_A_ mL^−1^ (8197–8768 µg_A_ mL^−1^ 95% confidence interval CI), and the WHO 20/136 SARS‐CoV‐2 reference had a binding value of 40 µg_A_ mL^−1^ (37–43 µg_A_ mL^−1^ 95% CI) to S1_HEK_ and 150 µg_A_ mL^−1^ (142–158 µg_A_ mL^−1^ 95% CI) to the Nucleocapsid*_E. coli_*. One mg_A_ IgG of the laboratory reference pool is equivalent to 11.13 IU of the WHO Ig reference and to the binding activity units (BAU), which are shown in Table 3.

**Table 2:** ELISA intra‐assay and inter‐assay coefficients of variation (CV) of the WHO references and study group's sera

	Parallelism		Repeatability	
	Samples/Repeats (*n*)	Mean Intra‐assay CV	Samples/Repeats (*n*)	Mean Inter‐assay CV
WHO 67/086	1/13	5.04%	1/13	5.57%
Spike‐S1_HEK_				
WHO 20/136	1/6	1.81%	1/7	7.06%
Values study group	120	7.12%	21/6	11.90%
High values	29	6.03%	7/6	14.00%
Medium values	37	3.96%	10/6	9.78%
Low values	54	9.88%	4/6	13.7%
Nucleocapsid*_E. coli_*				
WHO 20/136	1/7	3.07%	1/7	5.76%
Values study group	140	7.19%	21/6	6.62%
High values	67	6.50%	8/6	6.58%
Medium values	34	5.90%	9/6	5.97%
Low values	39	9.50%	4/6	8.19%
Nucleocapsid_HEK_				
Values study group	128	6.07		
High values	44	4.41%		
Medium values	50	6.50%		
Low values	34	7.58%		

**Table 3:** Conversion factors between the WHO standard units and IgG ELISA values

	IgG	IgG Pool	IgG_SARS‐CoV‐2_	IgG to S1_HEK_	IgG to N*_E. coli_*
	[IU mL^−1^]	[µg_A_ mL^−1^]	[BAU mL^−1^]	[µg_A_ mL^−1^]	[µg_A_ mL^−1^]
WHO 67/87	**94.4**	8482			
	1	89.9			
	11.13	1000			
WHO 20/136			**1000**	40	150
			6651		1000
			25056	1000	

Defined values of the WHO references are printed in bold. The other values are the means of repeated ELISA measurements and the converted values.

Furthermore, the results of the study groups' dilution series and the repeatability of the results of rerun samples were analysed (Table 2). The intra‐assay CV did not differ significantly between the different antigens and was at mean 6.80% (6.29–7.31% 95% CI) for the measurements to all three antigens in the study group. The variation among the low values (< 5 mg_A_ L^−1^) was significantly higher than that of the high or medium values.

With respect to repeatability, the medium values had the lowest CV, but the differences between high, median and low values were not significant. According to the European Medicines Agency, a CV of 15% or lower is acceptable for repeatability.^40^ The ELISAs clearly met the required repeatability. No experiments were performed to determine the repeatability of the Nucleocapsid_HEK_ ELISA.

### Clinical performance

**Figure 2:**
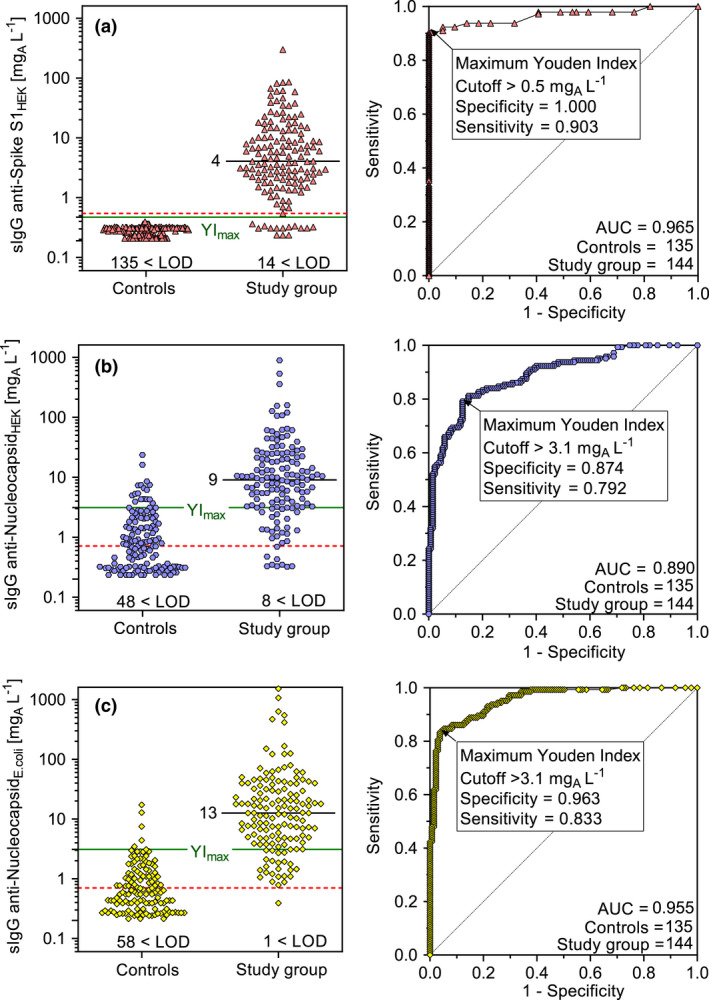
IgG results in the study and control groups. Specific IgG concentrations to the SARS‐CoV‐2 S1 antigen expressed in HEK cells **(a)**, to the nucleocapsid expressed in HEK cells **(b)** and to the nucleocapsid expressed in *E. coli* **(c)** are shown in the left panel, and the receiver operating curves (ROC) are shown in the right panel. In addition, the median values in the study group, the number of samples below the LOD (dashed line in plots), the area under the curve (AUC), the cut‐off for the maximum Youden index (YI_max_) and the sensitivity and specificity at the cut‐off value are all indicated.

The IgG results of the study group and the controls including ROC analyses are shown in Figure 2. The AUC for anti‐S1_HEK_ IgG, anti‐Nucleocapsid_HEK_ IgG and anti‐Nucleocapsid*_E. coli_* IgG was 0.965, 0.890 and 0.955, respectively. The cut‐off values for optimal discrimination between controls and the study group were estimated by the maximal YI (YI_max_), which were 0.903, 0.667 and 0.797 for anti‐S1_HEK_ IgG, anti‐Nucleocapsid_HEK_ IgG and anti‐Nucleocapsid*_E. coli_* IgG, respectively.

IgG values to nucleocapsids were higher in the study group as well as in the control group compared to the values in response to the Spike‐S1 antigen. The maxima reached 1522, 890 and 300 mg_A_ L^−1^, and the 90% quantiles were at 68, 55 and 35 mg_A_ L^−1^ for the Nucleocapsid*_E. coli_*, the Nucleocapsid_HEK_ and the Spike‐S1_Hek_, respectively. There were no significant differences in IgG concentrations between the subjects with symptoms and those where information on symptoms was lacking (data not shown).

For comparison, all sera from the study group participants were also tested using the semi‐quantitative IgG anti‐SARS‐CoV‐2 ELISAs from EuroImmun and the quantitative Spike‐S1 EliA FEIA from Thermo Fisher. The sensitivities of the immunoassays are shown in Table 4. The Spike‐S1_HEK_ ELISA had a similar sensitivity as the EuroImmun S1 ELISA, but a higher sensitivity than the Spike‐S1 EliA. The sensitivity of the Nucleocapsid*_E. coli_* was lower than that of the EuroImmun Nucleocapsid ELISA.

**Table 4:** Sensitivities of IPA ELISAs in comparison with commercial immunoassays

	Positives / Tested (*n*)	Sensitivity
Spike‐S1 EliA		
Cut‐off positives: U mL^−1^ > 10	110/144	76.4%
Cut‐off borderline: U mL^−1^ ≥ 7	122/144	84.7%
Spike‐S1 EuroImmun		
Cut‐off positives: ratio ≥ 1.1	126/144	87.5%
Cut‐off borderline: ratio ≥ 0.8	131/144	91.0%
Nucleocapsid EuroImmun		
Cut‐off positives: ratio ≥ 1.1	127/144	88.2%
Cut‐off borderline: ratio ≥ 0.8	129/144	89.6%
S1 or Nucleocapsid EuroImmun		
Cut‐off positives: ratio ≥ 1.1	138/144	95.2%
Cut‐off borderline: ratio ≥ 0.8	139/144	95.9%
Spike‐S1_HEK_ IPA positive		
Cut‐off positives: mg_A_ L^−1^ > 0.5	130/144	90.3%
Nucleocapsid*_E. coli_* IPA		
Cut‐off positives: mg_A_ L^−1^ > 3.1	120/144	83.3%
S1 or Nucleocapsid*_E. coli_* IPA	136/144	94.4%

**Figure 3:**
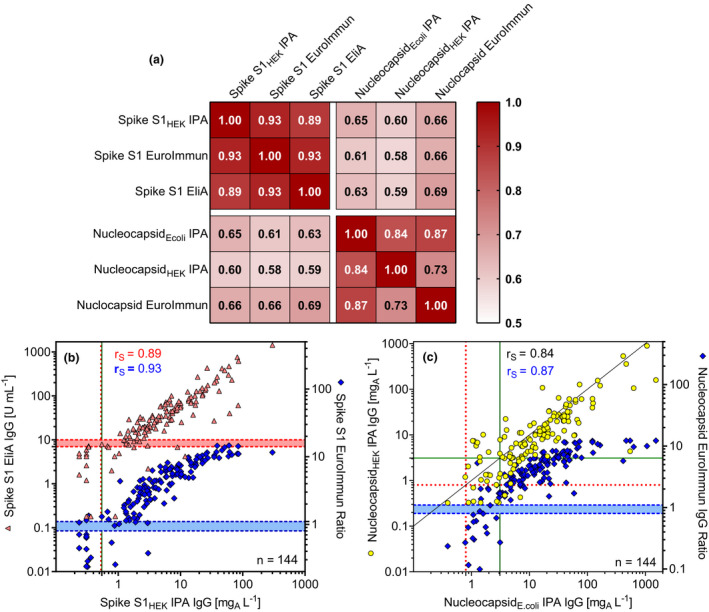
Correlations between immunoassay results and scatter plots. Heat map **(a)** of Spearman correlations (r_S_) between the study group's immunoassay results and scatter plots of Spike‐S1 immunoassay results **(b)** and nucleocapsid results **(c)**. The cut‐off values (see Table 4) for positivity and borderline results in EuroImmun ELISAs and in the Spike‐S1 EliA are indicated by dashed horizontal lines. The cut‐off values for the IPA ELISAs are shown with solid lines, the LODs are indicated using dotted lines, and the line of identity is shown for the Nucleocapsid ELISAs from IPA.

When the anti‐S1_HEK_ results were combined with the anti‐Nucleocapsid*_E. coli_* IgG results (positive if at least one test positive), sensitivity was 94.4% and specificity was 96.3%. Spearman correlations between all assay results in the study group were calculated, and scatter plots of the IgG values are shown in Figure 3. All assay results correlated highly significantly with one another. The correlation coefficients between anti‐S1 IgG results were in the range of 0.89–0.93 and between anti‐Nucleocapsid IgG results in the range of 0.73–0.87 (Figure 3a). Conversely, the correlation coefficients between S1 and Nucleocapsid IgG results were between 0.58 and 0.69 (Figure 3a). Scatter plots indicate that the log‐transformed values plotted against each other increased proportionally (Figure 3b and c); however, the semi‐quantitative EuroImmun ELISA became saturated at ratios around 10. In most cases above the cut‐off, the nucleocapsid expressed in *Escherichia coli* resulted in higher values than the nucleocapsid expressed in HEK cells (Figure 3c).

### Quantitative IgG results after vaccination

**Figure 4:**
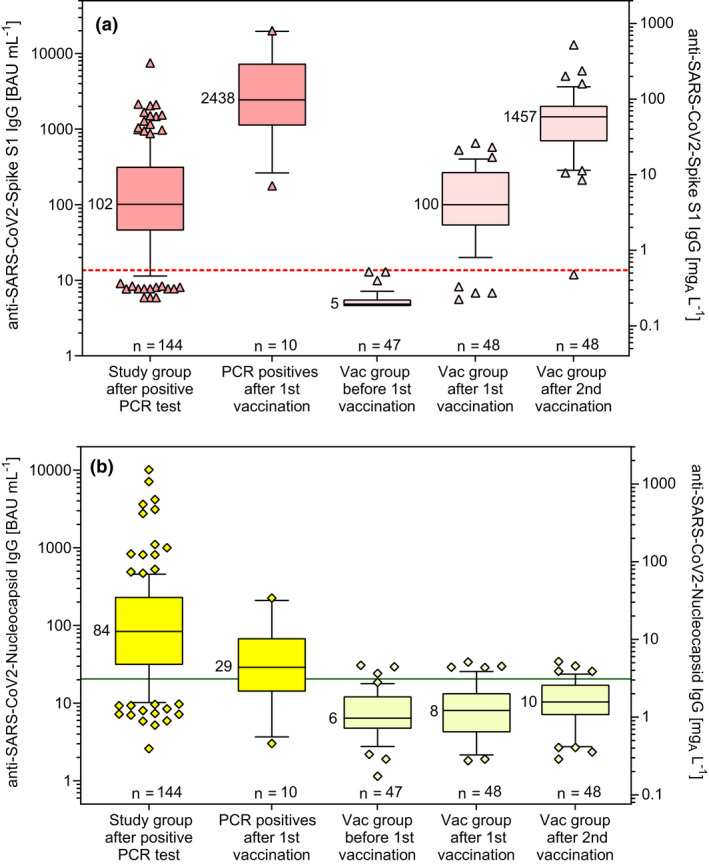
IgG results in vaccinated groups in comparison with those in the study group. Specific IgG concentrations to the SARS‐CoV‐2 S1 antigen expressed in HEK cells **(a)** and to the nucleocapsid expressed in *E. coli* **(b)**. Boxes comprise the 25^th^ and 75^th^ percentiles and the median values with numbers in BAU mL^−1^. Vertical bars show the 10^th^ and 90^th^ percentiles, and the symbols represent values outside of the given percentiles. The left axes have a scale in BAU mL^−1^, and the right axes show the corresponding scales in mg_A_ L^−1^. In addition, the number of tested samples, the LOD (dashed line) and the cut‐off for the maximum Youden index (solid line) are indicated.

The anti‐Nucleocapsid*_E. coli_* and anti‐Spike‐S1_HEK_ IgG ELISAs were used for quantification of antibodies after vaccination of 10 previously PCR‐positive individuals and a group of 48 volunteers without SARS‐CoV‐2 infection. The basic data of these groups are presented in Table 5. Only four people did not develop anti‐S1 antibodies after the 1^st^ vaccination and only one still remained negative after the 2^nd^ vaccination with BNT162b2. The IgG results were converted to BAU mL^−1^ by the factors shown in Table 3. IgG to S1 increased significantly after 1^st^ and 2^nd^ vaccination in the vaccinated group (Figure 4a) in contrast to IgG to the nucleocapsid which remained low (Figure 4b). On average, the anti‐Spike‐S1 IgG concentration was 23 times higher after the 2^nd^ vaccination than after the 1^st^ vaccination. Antibody concentrations tended to be lower with increasing age of the vaccinated. This effect was significant in the linear regression models of log‐transformed values as a function of age and more pronounced for anti‐Spike‐S1 IgG after the 1^st^ vaccination (*P*‐value = 0.0074) than after the 2^nd^ vaccination (*P*‐value = 0.0172). The 10 individuals vaccinated after SARS‐CoV‐2 infection had even higher mean and median anti‐Spike‐S1 IgG antibody concentrations than the vaccinated group after the second vaccination. The median IgG level of the 10 individuals after their positive PCR test had been 151 BAU mL^−1^ (range 13–2144 BAU mL^−1^) to Spike‐S1 and 129 BAU mL^−1^ (range 11–833 BAU mL^−1^) to the Nucleocapsid (data not shown).

**Table 5:** Baseline data from the vaccination group, which had no known COVID‐19 infection before vaccination, and the vaccinated previously infected individuals

	Vaccination group	PCR positives
	*n* = 48	*n* = 10
Age (years)	24–67 [Median = 50]	20–71 [Median = 57]
Gender		
Male (%)	31% (33/48)	30% (3/10)
Female (%)	69% (15/48)	70% (7/10)
Sample collection dates	2/2021–9/2021	2/2021–8/2021
SARS‐CoV‐2 PCR‐positive test	0%	100%
Symptomatic	0%	100%
Vaccine		
BNT162b2 BioNTech	100%	70% (7/10)
mRNA‐1273 Moderna		20% (2/10)
ChAdOx1 nCoV‐19 AstraZeneca		10% (1/10)
Days between PCR test and vaccination	–	91–401 [Median = 153]
Days after 1^st^ vaccination	13–33 [Median = 17]	14–46 [Median = 23]
Days between 1^st^ and 2^nd^ vaccination	21–43 [Median = 42]	–
Days after 2^nd^ vaccination	9–64 [Median = 21]	–

## Discussion

### Strategy to develop a quantitative and standardised ELISA for immunoglobulins

Quantification of the immune response to SARS‐CoV‐2 may be used as an indicator of immunity. Analysis of the T‐cell response or of neutralising antibodies requires specialised laboratories, sometimes also with increased safety measures, whereas the measurement of antibodies by ELISA is a standard method that can be performed in most immunological laboratories. In SARS‐CoV‐2 infections, different classes of immunoglobulins are produced almost simultaneously within the first weeks after infection.^17^, ^41^, ^42^, ^43^ Since the titres for IgG were the highest, longest lasting and proportional to the neutralising activity,^30^, ^31^, ^32^, ^41^ we restricted our ELISA development to this immunoglobulin class. Currently, there exist well‐characterised mAbs for human IgG that are optimal for standardised detection in immunoassays.^39^, ^44^ We combined two pan‐subclass‐IgG‐specific mAbs, with the Fd‐specific HP6045 as the capture antibody in the reference curve, and the Fc‐specific HP6017 for detection. Thus, the epitope for mAb binding on the human IgG antibodies should be distant from the paratopes for binding to SARS‐CoV‐2 epitopes in order to avoid steric hindrance. Using an internal serum pool with known total IgG concentrations for the reference curve, we obtained results in mg_A_ L^−1^. The ‘A’ in the index is intended to indicate that the IgG concentrations are antigen‐specific and depend on the avidity of IgG and affinity of mAbs. Strictly speaking, it is not the antibody concentration that is measured, but rather the antibody‐binding capacity. Because of the parallelism to the binding curve of the WHO reference for immunoglobulins, the IgG results can be converted into international units (IU) by multiplying mg_A_‐values with the factor 11.13. Conversely, 1 IU corresponds to 89.9 µg_A_ IgG (95% CI 86.8–92.9 µg_A_) and is close to the average value of 80.4 µg of isolated IgG present in the WHO reference preparation, which was determined with a 95% CI of 69.2–93.3 µg via a collaboration among 10 laboratories in 1972.^45^ By using the same standardised reference curve for different antigens, we could more easily compare the IgG concentration to the different SARS‐CoV‐2 antigens, as well as towards virus variants.

### Selection of SARS‐CoV‐2 antigens and assay performance

At the start of our project, we used the sequence of the original Wuhan virus, as well as the spike and nucleocapsid antigens, which were available at the time, as the basis to develop our ELISA method. However, SARS‐CoV‐2 antigen selection is one of the crucial aspects of assay development.^28^ Recombinant proteins are produced either by prokaryotic or by eukaryotic expression systems. To include post‐translational modifications, such as glycosylation or phosphorylation, we acquired recombinant S1 and nucleocapsid SARS‐CoV‐2 antigens that were expressed in a human cell line, which contained a His‐tag and were affinity‐purified. Mean intra‐assay CVs of 7% and inter‐assay CVs of 6–14% revealed parallelism to the IgG reference curve and high repeatability, respectively. However, the clinical performance by ROC analyses was better for the S1 ELISA (AUC = 0.965) compared to the Nucleocapsid_HEK_ ELISA (AUC = 0.890). Therefore, we used an additional antigen – a commercially available nucleocapsid that was expressed in *E. coli*. We observed improved clinical performance than in the Nucleocapsid_HEK_ ELISA with an AUC of 0.955, and a specificity of 96.3% and sensitivity of 83.3%. Importantly combining the anti‐S1_HEK_ and anti‐Nucleocapsid*_E. coli_*, IgG results improved sensitivity to 94.4%.

### Comparison of the ELISA with other immunoassays

The criterion for inclusion into our study group was a positive PCR test. In contrast, COVID‐19 patients including those who were hospitalised were selected for the positive collective in many other validation studies of antibody tests.^15^, ^16^, ^43^, ^46^ It was observed that severely ill patients had higher antibody titers,^7^, ^43^, ^47^, ^48^ while not all PCR‐positive patients developed an immune response.^49^ Consequently, a direct comparison of test sensitivities reported in the literature is of limited value. Therefore, we examined our study group with commercial IgG tests that were feasible with our laboratory equipment. These included semi‐quantitative ELISAs from the company EuroImmun, which were validated and used in several previous studies,^12^, ^15^, ^16^, ^32^, ^43^, ^48^, ^50^ and the quantitative FEIA from Thermo Fisher, which can be processed using the automated Phadia 250 system.

All test results correlated highly significantly with one another. In particular, the newly developed Spike‐S1 ELISA was equivalent or even superior to the commercial immunoassays in terms of clinical performance. In contrast, the Nucleocapsid*_E. coli_* ELISA did not quite reach the clinical sensitivity of the EuroImmun ELISA. A comparatively high non‐specific binding of the Nucleocapsid*_E. coli_* ELISA led to a significantly higher cut‐off value compared to the S1 ELISA, and the clinical specificity was also suboptimal. It is possible that minimal cross reactions to endemic human beta‐coronaviruses are responsible for the reactivity in the controls; alternately, the degree of purity of the recombinant protein was too low. However, it is known that a modified protein is used in the Euroimmun Nucleocapsid ELISA, and as a result, a high specificity could be achieved.^48^

While the results of the different immunoassays were highly correlative, the lack of agreement among the values was expected because of their different units. Only quantitative test systems, which are also traceable to international references, allow for comparability. This was addressed in recently published work in which IgG was measured using ImmunoCAP.^46^ The authors reported a median level of 34.7 µg mL^−1^ to the SARS‐CoV‐2 spike‐RBD and 24.5 µg mL^−1^ to a SARS‐CoV‐2 nucleocapsid (both proteins were expressed in HEK cells, biotinylated and bound to Streptavidin ImmunoCAPs) in 36 patients who were evaluated at a follow‐up subsequent to their hospital stay. These median values are higher than those obtained with the subjects in our study, which is plausible because of the generally more severe disease experienced by hospitalised patients. Other factors that can also significantly influence the results include the different antigens used and how they are bound to the solid surfaces. A comparison of five different commercial quantitative immunoassays to quantify IgG levels against the SARS‐CoV‐2 spike protein showed good overall agreement, but they were not interchangeable, even when converted to BAU mL^−1^ using the WHO international standard for SARS‐CoV‐2 immunoglobulin.^51^ According to the authors and in agreement with our results, this highlights the need for further standardisation of SARS‐CoV‐2 serology.

### Antibody concentrations after vaccination

The newly developed ELISAs were used to study antibody responses of 48 healthy volunteers before and after vaccination with BioNTech–Pfizer BNT162b2 vaccine. After 1^st^ and 2^nd^ vaccination, 92% and 98% of the participants had anti‐spike S1 IgG (medians 100 BAU mL^−1^ and 1457 BAU mL^−1^, respectively). Ten individuals previously infected with COVID‐19 developed even higher antibody concentrations after vaccination (median 2438 BAU mL^−1^). Similar strong immune responses after vaccination with BNT162b2 have also been published previously.^52^, ^53^ A limitation of our results after vaccination is the small number of samples, especially in the previously infected group.

A robust correlation was seen between binding antibody titres and efficacy across different vaccines.^54^ Initial correlates of antibody concentrations sufficient for protection against COVID‐19 were reported^55^: The anti‐spike IgG level associated with 80% vaccine efficacy against primary symptomatic SARS‐CoV‐2 infection during a 4‐ to 6‐month period was estimated to be 264 BAU mL^−1^. However, the analyses by Feng *et al*.^55^ had been conducted on samples taken 28 days after two doses of Oxford–AstraZeneca ChAdOx1 nCoV‐19 and might not apply to protection afforded by the BNT162b2 vaccine used in our study.

## Conclusions

The ELISA method for the quantification of specific human IgG showed good intra‐ and inter‐assay reproducibility and parallelism to international reference sera. Furthermore, the clinical performance with the S1 spike protein expressed in HEK cells was not inferior to commercial test systems. However, improvements are required for the Nucleocapsid ELISA. One advantage of the ELISA method presented here is that adaptation to new antigens, for example mutated variants of the spike protein, can be easily implemented. In contrast to other methods for antibody measurements against SARS‐CoV‐2,^56^, ^57^ no SARS‐CoV‐2‐specific components are used apart from the SARS‐CoV‐2 antigen. In addition, the instruments used here are all part of the standard equipment available in most immunological laboratories, which makes the method suitable for many research laboratories.

In order to directly compare values from different laboratories, however, standardisation of the SARS‐CoV‐2 antigens is necessary, as is the use of international reference sera. The immunoassays could then be used to determine vaccine‐dependent estimates of protective antibody concentrations, which will then represent a simpler approximation compared to neutralising antibodies.

## Methods

### Human subjects

The study was carried out in accordance with the Code of Ethics of the World Medical Association (Declaration of Helsinki) for experiments involving humans and was approved by the local Ethics Committee of the Ruhr University Bochum in Germany (registration no. 20‐7007, 2020‐09‐04 and amendment, 2021‐02‐01). The study group included individuals with previously documented SARS‐CoV‐2 infection who were willing to provide blood samples and participate in our study. The vaccination groups consisted of employees of our institute and their relatives and friends who voluntarily donated sera before and after their vaccinations. The following vaccines had been used: BNT162b2 (BioNTech, Mainz, Germany), mRNA‐1273 (Moderna Cambridge, MA, USA) and ChAdOx1 nCoV‐19 (AstraZeneca, Cambridge, UK). Sera were obtained as anonymised samples according to data protection guidelines via a trustee from the following institutions: BG University Hospital Bergmannsheil Bochum, Practice for Laboratory Medicine and Microbiology Bochum (Dr Biermann‐Göcke), Specialist Practice Professor Dr BL Herrmann Bochum and IPA Bochum. Samples of the study group were classified as ‘true positives’ if a positive PCR test was recorded 14–100 days prior to the submission of the blood sample.

For the negative controls, pre‐pandemic sera from previous research projects (April 2006–December 2018) were used with approval of the Ethic Committee of the Ruhr University Bochum in Germany (registration number 1563, 2009‐09‐20, and 17‐6022, 2017‐07‐04). The participants provided informed consent for the use of their sera in further research projects to determine biomarkers, including antibodies.

Fifty sera samples from individuals (age 19–64, mean 42 years), collected between July 2006 and September 2007, were pooled, frozen in aliquots at −20°C and used as an internal laboratory reference. The total IgG concentration of this serum pool was previously measured by an external accredited medical laboratory and reported as 11.6 mg mL^−1^ (6.17 mg mL^−1^ IgG1, 4.38 mg mL^−1^ IgG2, 0.67 mg mL^−1^ IgG3 and 0.38 mg mL^−1^ IgG4).

### SARS‐CoV‐2 antigens

Recombinant SARS‐CoV‐2 S1 protein (Val16‐Arg685 from NCBI sequence: YP_009724390.1) with a poly‐histidine tag, expressed in human embryonic kidney 293 (HEK‐293) cells, was purchased from Sino Biological, Eschborn, Germany (40591‐V08H); recombinant SARS‐CoV‐2 nucleocapsid (N) protein (Met1‐Ala419 from GenBank sequence QHD43423) with a C‐terminal poly‐histidine tag expressed in HEK‐293 cells was purchased from RayBiotech (Peachtree Corners, GA, USA, 230‐30164); and recombinant SARS‐CoV‐2 nucleocapsid (N) protein (Met1‐Ala419 from GenBank sequence QHD43423) with a C‐terminal serine and poly‐histidine tag expressed in *E. coli* cells was purchased from Trenzyme, Konstanz, Germany (P2020).

### SARS‐CoV‐2 ELISAs

The following commercial anti‐SARS‐CoV‐2 IgG immunoassays were used according to the manufacturers' instruction: anti‐S1 IgG ELISA (Anti‐SARS‐CoV‐2, EI 2606‐9601 G; Euroimmun), anti‐Nucleocapsid IgG ELISA (Anti‐SARS‐CoV‐2‐NCP, EI 2606‐9620‐2 G, Euroimmun) and EliA SARS‐CoV‐2‐Sp1 IgG Fluorescence Enzyme Immunoassay (FEIA) on Phadia 250 instrument (Thermo Fisher Scientific, Freiburg, Germany). If the sample concentrations exceeded the range of the EliA assay, then the samples were manually pre‐diluted at 1:10.

For the IPA‐established ELISAs, three strips (F8) of Nunc 96‐well Flat MaxiSorp Immunoplates (Thermo Fisher Scientific) were coated with 100 µL per well (400 ng per well) of the pan‐anti‐human‐FD‐IgG monoclonal antibody HP6045 (05‐4500, Thermo Fisher Scientific) for the calibration curve, and a further nine strips were coated with 100 µL of the SARS‐CoV‐2 antigen (250–400 ng per well) in 0.1 m carbonate–bicarbonate buffer pH 9.6 overnight at 4°C. On the following day, plates were blocked with 1% Tween‐20 in phosphate‐buffered saline (PBS) for 1 h at 22°C. The calibration curve was obtained by adding 100 µL of 10 serial 3/5 dilutions of the internal laboratory reference pool serum with IgG concentrations ranging from 5.85 to 580 ng mL^−1^ to the wells coated with anti‐human IgG antibody. Each serum sample was tested using three serial 3/5 dilutions, which were added to the wells with the SARS‐CoV‐2 antigens. The start sample was diluted 1:100 using PBS with 0.05% Tween‐20 (PBST) for both the references and samples. Only in the case of the SARS‐CoV‐2 nucleocapsid expressed in *E. coli*, the sample and reference dilution buffer additionally contained 20 µg mL^−1^ *E. coli* protein to reduce unspecific binding. After incubation for 1 h at 22°C, the bound human IgG was detected with 100 µL biotinylated pan‐anti‐human‐Fc‐IgG antibody HP6017 (05‐4240, Thermo Fisher Scientific) diluted 1:1000 in PBST, followed by 100 µL/well of streptavidin–peroxidase conjugate (S5512, Sigma, Steinheim, Germany; diluted 1/20 000 with PBST) for 1 h at 22°C and finally 100 µL ABTS substrate [2,20‐azinobis(3‐ethylbenzothiazoline‐6‐sulfonic acid) diammonium salt; Sigma] in 50 mm phosphate–citrate buffer, pH 4.2, with 0.015% hydrogen peroxide. The reaction was stopped with 0.32% sodium fluoride, and the absorbance was read at 414 nm in a SpectraMax M2 (Molecular Devices, Sunnyvale, CA, USA). Sample concentrations were calculated by interpolation of optical density (OD) values on a four‐parameter fitted reference curve using Softmax Pro 7.0.3 (Molecular Devices) and reported as ng_A_ mL^−1^. The lower limit of detection (LOD) was the concentration corresponding to OD_414_ = 0.05 above the minimal value of the four‐parameter curve fit function (parameter A) which corresponds to a mean of 5 ng_A_ mL^−1^. The upper limit depended on the linear range of the sigmoidal curve corresponding at mean to 340 ng_A_ mL^−1^. In case, two or more of the sample dilutions were above the upper detection limit, and then, the sample was rerun using higher dilutions. The measurements were also repeated if the coefficient of variation of the calculated concentrations of the sample dilutions in range was greater than 25%.

### Parallelism, repeatability and standardisation

To investigate parallelism between the IgG calibration curve and the sample dilutions, the intra‐assay CV value of the sample dilution series was calculated for the newly developed ELISAs. Samples with results > 15 mg_A_ L^−1^ were considered ‘high’, results > 5 and ≤ 15 mg_A_ L^−1^ were considered ‘medium’, and results ≤ 5 mg_A_ L^−1^ were considered ‘low’. To investigate the repeatability of the Spike‐S1_HEK_ IPA and Nucleocapsid*_E. coli_* IPA ELISA, 21 samples with previously high, medium and low results were repeatedly tested on different plates and days and the mean inter‐assay CV was calculated.

For standardisation, the WHO international standard for human immunoglobulin G, A, M^36^ and the first WHO international standard anti‐SARS‐CoV‐2 for human immunoglobulin^37^ [67/086 and 20/136, respectively, National Institute for Biological Standards and Control (NIBSC), Hertfordshire, UK] were used. The solution for the former (WHO 67/086) was prepared by dissolving the contents of one ampoule in 1 mL of distilled water with a resulting concentration of 94.4 IU mL^−1^,^36^ and the WHO 20/136 solution was prepared by dissolving the contents of one ampoule in 250 µL distilled water with a reported resulting concentration of 1000 BAU mL^−1^ with regard to binding to SARS‐CoV‐2 antigens.^37^

Parallelism and repeatability were tested as described above, and converting factors between mg_A_ L^−1^ to IU L^−1^ and BAU L^−1^ were calculated.

### Statistical methods

Statistical analyses using Mann–Whitney and Kruskal–Wallis tests, two‐way ANOVA with Bonferroni multiple comparison, calculation of confidence intervals (CI) and Spearman correlations, linear regression of log‐transformed values and ROC analyses were performed with GraphPad Prism 9 (GraphPad Software, San Diego, CA, USA). For ROC, correlation analyses and graphs, IgG values less than the detection limit were set to 2/3 of this limit. The Youden index (YI = Sensitivity + Specificity − 1) was calculated using Excel (Office 2019, Microsoft, München, Germany).

## Conflict of Interest

The authors declare that they have no conflict of interest to disclose in the preparation of this manuscript.

## Author Contributions

**Ingrid Sander:** Conceptualization; Data curation; Formal analysis; Investigation; Methodology; Project administration; Software; Validation; Visualization; Writing – original draft; Writing – review & editing. **Sabine Kespohl:** Conceptualization; Data curation; Investigation; Methodology; Project administration; Resources; Validation; Writing – original draft; Writing – review & editing. **Eva Zahradnik:** Investigation; Methodology; Validation; Writing – original draft; Writing – review & editing. **Philipp Göcke:** Data curation; Investigation; Resources; Validation; Writing – review & editing. **Ingolf Hosbach:** Investigation; Resources; Writing – review & editing. **Burkhard L Herrmann:** Data curation; Investigation; Resources; Writing – review & editing. **Thomas Brüning:** Funding acquisition; Resources; Supervision; Writing – review & editing. **Monika Raulf:** Conceptualization; Funding acquisition; Methodology; Project administration; Resources; Supervision; Writing – review & editing.
